# Application of Breast Ultrasound Elastography to Differentiate Intracapsular Collection from Silicone-Induced Granuloma of Breast Implant Capsule Complementarily to Contrast-Enhanced Breast Magnetic Resonance Imaging

**DOI:** 10.1177/1178223417737994

**Published:** 2017-11-02

**Authors:** Eduardo de Faria Castro Fleury, Ana Claudia Gianini, Veronica Ayres, Luciana C Ramalho, Decio Roveda, Vilmar Marques de Oliveira

**Affiliations:** 1 Department of Radiology, IBCC—Insituto Brasileiro de Controle do Câncer, São Paulo, Brazil; 2 Department of Radiology, Faculdade de Ciências Médicas da Santa Casa de São Paulo, Santa Casa de Misericórdia de São Paulo, São Paulo, Brazil

**Keywords:** Anaplastic large-cell lymphoma, elastography, NMR imaging, seroma

## Abstract

**Background:**

To determine whether there is correlation between magnetic resonance imaging (MRI) findings and breast elastography to differentiate seroma/hematoma from silicone-induced granuloma of breast implant capsule (SIGBIC).

**Methods:**

Prospective study of 99 patients with breast implants submitted to breast MRI during the period from February 1 to May 1, 2017. Patients who present MRI findings of seroma/hematoma or SIGBIC were submitted to a complementary ultrasound elastography study to evaluate the correlation of the results. The criteria adopted for the diagnosis of granuloma by MRI were heterogeneous hypersignal in the T2-weighted sequences, late contrast enhancement, and black drop sign. Lesions that did not enhance after the use of contrast were considered as seroma/hematoma. By elastography, the results were considered positive for granuloma when presented as hard lesions, whereas seroma/hematoma presented as soft lesions.

**Results:**

Of the 99 patients evaluated, 15 were included in the study. Of the 15 patients, 9 had solid intracapsular MRI masses, whereas 6 presented collections without contrast enhancement. The complementary elastography study showed correlation with MRI results in all cases of SIGBIC and seroma/hematoma, being elastography able to differentiate lesions from solid to cystic.

**Conclusions:**

Elastography of intracapsular masses in breast implants presented results compatible with those found by MRI to differentiate solid lesions from collections.

## Background

Silicone-induced granuloma of breast implant capsule (SIGBIC) has recently been described as mass formation within the fibrous capsules of breast implants. It is believed to be an underdiagnosed entity as there are few reports in the literature. It results from the formation of granuloma, induced by silicone bleeding from the inside of the intact breast implant into the space between the implant and its fibrous capsule.^1^

The intracapsular seroma/hematoma is a well-known complication, with well-defined diagnostic criteria. It consists of exudate between the fibrous capsule and the implant, which can be spontaneously reabsorbed. When it occurs 12 months after surgery, it is considered late seroma, whereas the late hematoma is due to repeated microfractures of the fibrous capsule.^2^

These 2 entities have similar MRI presentation without contrast media, where the differentiation between SIGBIC and seroma is practically impossible. For differentiation, it is imperative to use intravenous contrast.^3^

One of the first and main applications of diagnostic ultrasonography would be to differentiate between solid and cystic lesions. However, cysts with thick content are difficult to differentiate from solid masses. Complementary Doppler flowmeter study can be used as a diagnostic aid; however, it requires the lesion of interest to be hypervascularized.^4^

Nowadays, the elastography tool in ultrasound devices is increasingly available, allowing the determination of the stiffness of the lesion by means of external compression or shear wave. This tool has shown important role in the differentiation of breast masses, especially solid masses and complicated cysts.

The purpose of this short communication is to verify whether there is correlation of the MRI findings with the elastography to differentiate SIGBIC from seroma.

## Materials and Methods

**Figure 1.**
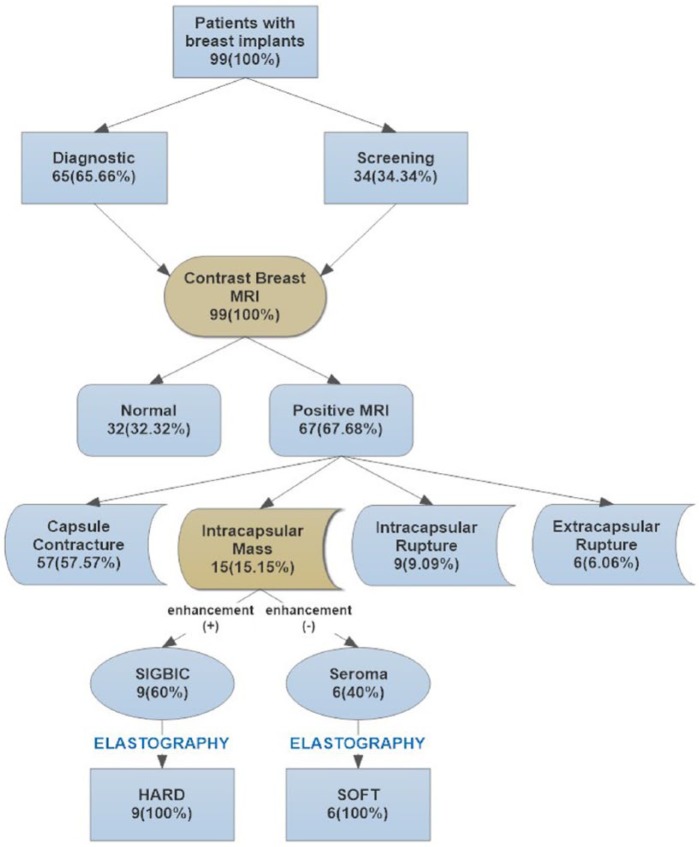
Flowchart for SIGBIC diagnosis. MRI indicates magnetic resonance imaging; SIGBIC, silicone-induced granuloma of breast implants capsule.

We prospectively evaluated breast magnetic resonance imaging (MRI) scans from 99 patients with breast implants from February 2017 to May 2017. The consecutive examinations were from women who were referred for MRI scans to our service, being diagnostic when they had clinical symptoms or for screening when they had a higher risk for development of breast cancer. Patients with intracapsular collection or patients with intracapsular mass, both with intact breast implants, were included in the study (Figure 1). Patients with intracapsular or extracapsular rupture of breast implants or with isolated capsular contracture findings at MRI were excluded.

Patients included in the study were recalled for performing elastography. A specialist in the method who has 9 years of experience in elastography performed the study of elastography directed to the MRI findings. The elastographic images were classified as soft, intermediate, and hard as proposed in a previous study.^5^

The study was conducted in accordance with local ethics committee regulations.

## Differentiation Between SIGBIC and Seroma by MRI

The presentation of SIGBIC and seroma by MRI is very similar in the noncontrast phases. For the differential diagnosis, it is imperative to administer intravenous contrast media. The MRI findings that allow the diagnosis of granuloma consist of an expansive lesion that determines a compressive effect on the breast implant, with heterogeneous hypersignal in the T2-weighted sequences. The enhancement is usually late, more evident in the last acquisitions of the dynamic phase (>4 minutes). Usually, the contrast is from the periphery to the center of the lesion. It also may present the black drop sign, which is a focus of a marked hyposignal at the interface between the implant and the granuloma. These findings were described in a recent study.^4^

The seroma will manifest as an expansive lesion with similar heterogeneous hypersignal in T2-weighted sequences. However, in the dynamic phases, no areas of intracapsular enhancement will be observed.

## Differentiation Between SIGBIC and Seroma by elastography

**Figure 2.**
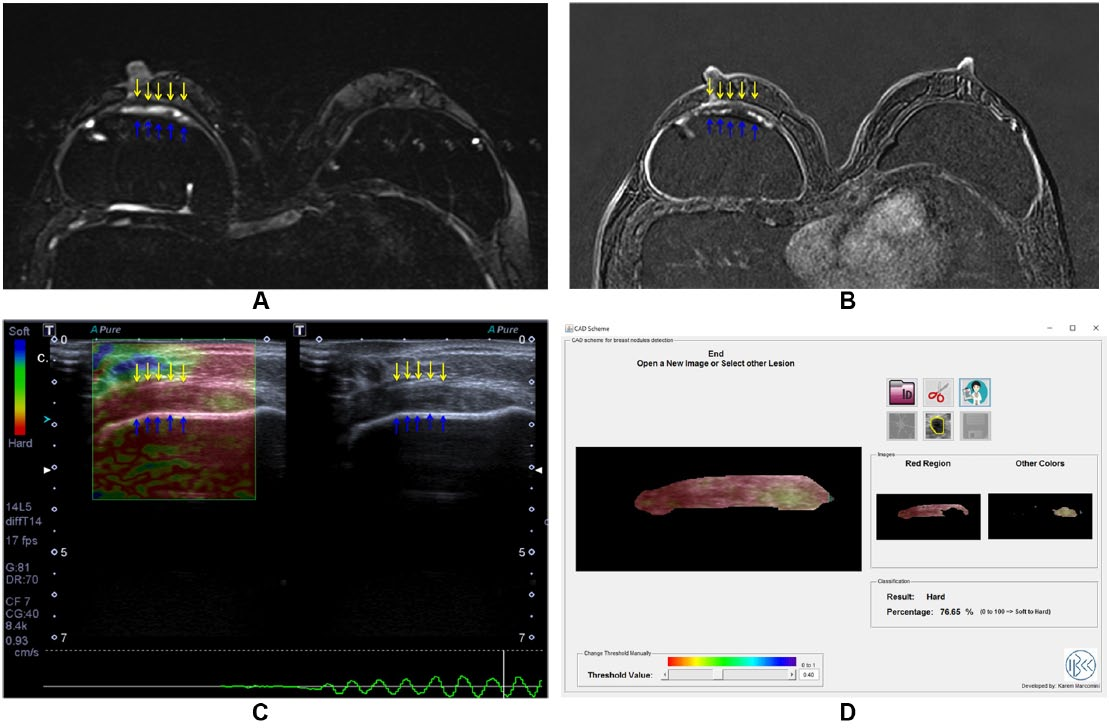
A 58-year-old woman with breast augmentation surgery 10 years ago. (A) Axial T2-weighted sequence with silicone suppression presents collection between the fibrous capsule (yellow arrows) and the elastomer of the implant (blue arrows). (B) In the dynamic sequence, there is enhancement of this collection corresponding to solid content. (C) By elastography, it is observed to correspond to hard lesion. (D) Elastography report.

**Figure 3.**
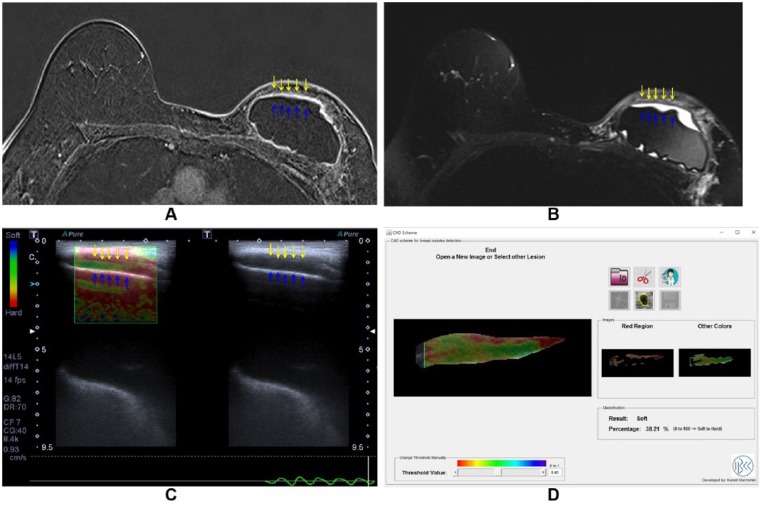
A 42-year-old woman breast augmentation surgery 3 years ago. (A) Axial T2-weighted sequence with silicone suppression presents collection between the fibrous capsule (yellow arrows) and the elastomer of the implant (blue arrows). (B) In the dynamic sequence, there is no enhancement of this collection corresponding to fluid collection. (C) By elastography, it is observed to correspond to soft lesion. (D) Elastography report.

Both granuloma and seroma will present as a thick collection at conventional ultrasonography. Because intracapsular granulomas are poorly vascularized, the Doppler flowmetry data do not generally contribute to the differential diagnosis. As the elastography evaluates the stiffness of the area of interest, the granulomas will present as hard lesions, whereas the seromas will present as soft lesions (Figures 2 and 3).

## Comparison Between MRI and Elastographic findings

The MRI findings of the lesions were compared with those obtained by the elastographic study. We evaluated whether there was correlation between the findings by the 2 methods.

## Results

Of the 99 patients evaluated, 9 (9.1%) with SIGBIC and 6 (6.1%) with seromas were included. Of the 9 patients with SIGBIC, 6 were confirmed by surgical excision. Of the 6 patients with seroma, 2 were drained and 4 were being followed up.

By MRI, all 6 (100%) patients with seromas presented with heterogeneous hypersignal in the T2-weighted sequences, with no contrast enhancement. All seromas were associated with capsular contracture. The mean implant placement time was 1.7 years, with a median of 1 years and a standard deviation of 1.0 for seromas.

All patients with SIGBIC presented expansive mass with heterogeneous hypersignal at T2, with late contrast enhancement on MRI. All patients had the black drop signal associated. The mean time of placement of the implants was 10.2 years, with a median of 7 years and a standard deviation of 11.8 years. Of the 9 patients diagnosed with granuloma, 7 (77.8%) had associated capsular contracture and 1 (1.1%) had SIGBIC associated with seroma, being considered as SIGBIC given its relevance (Table 1).

**Table 1.** Case distribution according to the surgical time (surg.).

Case	Surg.	MRI findings				Elastography		Result
		Mass	T2	C^+^	BDS	Hard	Soft	
1	1	+	+	−	−	−	+	Seroma
2	3	+	+	+	+	+	−	SIGBIC
3	17	+	+	+	+	+	−	SIGBIC
4	40	+	+	+	+	+	−	SIGBIC + seroma
5	10	+	+	+	+	+	−	SIGBIC
6	3	+	+	−	−	−	+	Seroma
7	1	+	+	−	−	−	+	Seroma
8	3	+	+	−	−	−	+	Seroma
9	7	+	+	+	+	+	−	SIGBIC
10	12	+	+	+	+	+	−	SIGBIC
11	1	+	+	+	+	+	−	SIGBIC
12	1	+	+	+	+	+	−	SIGBIC
13	1	+	+	−	−	−	+	Seroma
14	1	+	+	−	−	−	+	Seroma
15	1	+	+	+	+	+	−	SIGBIC

Description of magnetic resonance imaging findings (MRI findings): presence of mass (MASS), hypersignal in the T2-weighted sequence (T2), contrast enhancement (C^+^) and black drop sign (BDS), and correlation with elastography as soft or hard lesion.

Elastography could differentiate granulomas from seromas in all 15 (100%) cases. All granulomas presented as hard lesions, whereas all seromas presented as soft lesions at the elastography study.

## Discussion

Breast MRI scans are increasingly performed to evaluate complications of breast implants. Much attention has recently been dispensed due to the reports of anaplastic large-cell lymphoma (ALCL) related to silicone implants. Recent studies describe as the main complications of the implants the formation of seromas/hematomas (early or late), capsule contractures, and infections.^6^ The criteria adopted to describe capsular contracture are thickening of the fibrous capsule with contrast enhancement associated with morphological changes of the breast implant and clinical findings. Recently, the presence of intracapsular granuloma in intact breast implants has been described.

Most often, the breast MRI scan is performed without the postcontrast phase, which limits the evaluation of any proliferative mass inside the fibrous capsules. As MRI findings of seromas are very similar to those of granulomas, we believe that granuloma may be underdiagnosed in clinical practice.

The diagnosis of SIGBIC is of fundamental importance because it is due to extravasation of silicone particles in intact breast implant. This silicone overflow is extremely toxic when it meets the fibrous capsule of the implant and triggers the formation of silicone-induced granuloma. As the fibrous capsule of the implant works as a physical barrier that protects the rest of the body from the contents inside the implants, there are usually no systemic repercussions of this complication. However, if there is a breakdown of this barrier, systemic changes may manifest as the silicone implant incompatibility syndrome.^7^ In these cases, percutaneous biopsy is contraindicated given the possibility of breaking the physiological barrier and exposure of the intracapsular contents. Studies still need to be performed to determine whether there is relationship between SIGBIC and ALCL.^1^ We hypothesized that ALCL is an evolution of SIGBIC, an autoimmune disorder. This should be confirmed in future studies.

Our results indicate that elastography can differentiate between intracapsular seroma and granuloma in the implants capsule. It consists of a low cost and complexity study, widely available, with no side effects. It may be performed as a complementary MRI study, especially in cases where MRI contrast media is contraindicated.

In the histologic results, the granulomas appear as extremely rigid masses, of slow growth and of low local aggressiveness. It is interesting to note, by our data, that seromas are related to more recent surgeries, whereas SIGBIC is related to older surgeries. Whenever late lesions appear in breast implants capsule, SIGBIC should be suspected and MRI should be performed with contrast. Elastography can be used in indeterminate cases by MRI as a complementary study for better diagnostic elucidation.

If we only consider the findings of the MRI noncontrast phase, all the 15 (15.15%) lesions were found to present as expansive lesions with heterogeneous hypersignal in the T2-weighted sequences. Usually, these lesions are considered as seroma/hematoma without contrast media injection. However, when contrast is injected, our data show that most of them are granulomas, probably underdiagnosed in previous studies. The criteria for the diagnosis of SIGBIC by MRI are very strict, which favors its high diagnostic accuracy. We believe that the number of SIGBIC cases may be underestimated by this study if less stringent criteria were used.

Our study is an initial study, the results of which must be confirmed by increasing the sample and performing other multicenter studies to replicate our results. However, it is important to alert the presence of SIGBIC so that more attention must be given to this diagnosis.

## Conclusions

Our results demonstrate there is correspondence between breast MRI and elastography to the differentiation SIGBIC from seroma. Elastography can be used as a complementary method to MRI, especially in patients where contrast injection is contraindicated.
